# Fast and Accurate Microplate Method (Biolog MT2) for Detection of *Fusarium* Fungicides Resistance/Sensitivity

**DOI:** 10.3389/fmicb.2016.00489

**Published:** 2016-04-06

**Authors:** Magdalena Frąc, Agata Gryta, Karolina Oszust, Natalia Kotowicz

**Affiliations:** Laboratory of Molecular and Environmental Microbiology, Department of Soil and Plant System, Institute of Agrophysics, Polish Academy of SciencesLublin, Poland

**Keywords:** *Fusarium* fungicides resistance, MT2 microplates, hole-plate method, chemical sensitivity, fungicides, phenomic profiles

## Abstract

The need for finding fungicides against *Fusarium* is a key step in the chemical plant protection and using appropriate chemical agents. Existing, conventional methods of evaluation of *Fusarium* isolates resistance to fungicides are costly, time-consuming and potentially environmentally harmful due to usage of high amounts of potentially toxic chemicals. Therefore, the development of fast, accurate and effective detection methods for *Fusarium* resistance to fungicides is urgently required. MT2 microplates (Biolog^TM^) method is traditionally used for bacteria identification and the evaluation of their ability to utilize different carbon substrates. However, to the best of our knowledge, there is no reports concerning the use of this technical tool to determine fungicides resistance of the *Fusarium* isolates. For this reason, the objectives of this study are to develop a fast method for *Fusarium* resistance to fungicides detection and to validate the effectiveness approach between both traditional hole-plate and MT2 microplates assays. In presented study MT2 microplate-based assay was evaluated for potential use as an alternative resistance detection method. This was carried out using three commercially available fungicides, containing following active substances: triazoles (tebuconazole), benzimidazoles (carbendazim) and strobilurins (azoxystrobin), in six concentrations (0, 0.0005, 0.005, 0.05, 0.1, 0.2%), for nine selected *Fusarium* isolates. In this study, the particular concentrations of each fungicides was loaded into MT2 microplate wells. The wells were inoculated with the *Fusarium* mycelium suspended in PM4-IF inoculating fluid. Before inoculation the suspension was standardized for each isolates into 75% of transmittance. Traditional hole-plate method was used as a control assay. The fungicides concentrations in control method were the following: 0, 0.0005, 0.005, 0.05, 0.5, 1, 2, 5, 10, 25, and 50%. Strong relationships between MT2 microplate and traditional hole-plate methods were observed regarding to the detection of *Fusarium* resistance to various fungicides and their concentrations. The tebuconazole was most potent, providing increased efficiency in the growth inhibition of all tested isolates. Almost all among tested isolates were resistant to azoxystrobin-based fungicide. Overall, the MT2 microplates method was effective and timesaving, alternative method for determining *Fusarium* resistance/sensitivity to fungicides, compering to traditional hole-plate approach.

## Introduction

The genus *Fusarium* contains over 70 cosmopolitan species, occurring in natural conditions in different regions of the world. They are common in soil, as saprophytes, but they can also grow on plant residues and other organic substances (Kotowicz et al., 2014). *Fusarium* species may cause plant diseases of both parts underground and aboveground (Leslie and Summerell, 2006). The application of fungicides plays an important role in *Fusarium* diseases management (Amarasinghe et al., 2013). Fungicides application is one of the available measurement that may indirectly contribute reduce the risk of pathogen infection, however, it does not guarantee getting rid of the pathogen (Baturo-Cieśniewska et al., 2011).

In general, sterole biosynthesis inhibitors, which includes triazoles fungicides are reported to be the most effective chemicals against *Fusarium* ssp. (Pirgozliev et al., 2002; Amarasinghe et al., 2013). The increasing usage of triazoles in Europe and Asia leads to selection of less sensitive *Fusarium* isolates (Klix et al., 2007; Yin et al., 2009; Becher et al., 2010), which is considered as a key determinant of the relatively low fungicide efficacy in the field conditions (Spolti et al., 2012). The strobilurins can cause increase of mycotoxin deoxynivalenol accumulation (Haidukowski et al., 2005). However, the carbendazim resistance of *Fusarium* can cause diseases even after 2–3 years from first fungicide application (Zhou and Wang, 2001). Therefore, the efficacy of fungicide use for the control of *Fusarium* diseases and mycotoxins production varies from being highly effective to even increasing mycotoxins levels (Mullenborn et al., 2008). Strobilurin fungicides block electron flow through one of the protein complexes, and disrupt energy supply. Several pathogens have quickly developed resistance to strobilurins. The reason of this resistance can be an alternative oxidase (AOX) which is a strobilurin-insensitive terminal oxidase that allows electrons from ubiquinol to bypass appropriate protein complex. Its synthesis is constitutive in some fungi but in many others is induced by inhibition of the main pathway. Salicylhydroxamic acid (SHAM) is a characteristic inhibitor of AOX, and several studies have explored the potentiation of strobilurin activity by SHAM (Wood and Hollomon, 2003).

The need for finding fungicides against *Fusarium* and monitor of their sensitivity is a key step in the chemical plant protection and using appropriate chemical agents by farmers. For these reasons the rapid, accurate method for evaluation of *Fusarium* fungicides resistance in monitoring study is critical. Existing conventional methods of qualitative and quantitative evaluation of fungal isolates resistance to fungicides are costly, time-consuming and even more they could be environmentally harmful due to usage of high amounts of potentially toxic chemicals (Arikan, 2007; Rekanović et al., 2010; Panek et al., 2016). Therefore, the development of fast, accurate and effective *Fusarium* fungicides resistance detection method is urgently required. MT2 microplates method is traditionally used for bacteria identification and the evaluation of their ability to utilize different carbon substrates (Kadali et al., 2012). This system was also successfully used as a method in fast screening of native lignocellulosic-straw-degrading bacteria (Taha et al., 2015), identifying of bacteria, which are able to decompose hydrocarbon fractions (Kadali et al., 2012) and metabolize microcystin-LR (Manage et al., 2009). However, to the best of our knowledge, there are no reports concerning the use of this technique to determine fungicides resistance of the *Fusarium* isolates. Because the wells of the MT2 microplate do not contain any carbon sources, they can be loaded by any set of carbon sources or their mixtures into the wells. Therefore the MT2 microplates can be designed and optimized as approach for different applications, including study of fungi. Additionally, each well already contains the buffered nutrient medium and the tetrazolium chemistry suitable for growth of wide range of microorganisms (Bochner, 1989). In recent years, there has been exploration of several alternative nutrient sources and nutrient media for fungal culture (Basu et al., 2015). Therefore, the nutrient medium in MT2 plates can be also used for fungal growth after implementation with other components and inoculating fluids. Tetrazolium violet is used as a redox dye to colorimetrically indicate utilization of the carbon sources. However, the reading can also include optical density measurement without color development analysis, which can be used for fungal growth intensity evaluation instead of colorimetric measurement, allowing optimizing MT2 microplates for these organisms.

Hence, the objectives of this study were to: (1) develop a fast *Fusarium* fungicides resistance detection method; (2) compare the approach between both traditional hole-plate method and MT2 microplate-based assay; (3) evaluate the efficacy of three different fungicides against nine *Fusarium* isolates; (4) determine relationship between *Fusarium* fungicide sensitivity and metabolic profile of the tested isolates.

## Materials and Methods

### Fungal Strains and Growth Conditions

Nine *Fusarium* strains (G15/14, G17/14, G18/14, G21/14, G22/14, G24/14, G25/14, G27/14, and G29/14) were selected among fungal collection of Laboratory of Molecular and Environmental Microbiology, Institute of Agrophysics Polish Academy of Sciences (Lublin, Poland). Fungal strains were isolated from soil (G15/14, G21/14, G22/14, G25/14, G27/14) and wheat (G17/14, G18/14, G24/14, G29/14) in eastern Poland (N 50°59′, E 23°08′). Fungal strains were isolated from soil by selective Bengal Rose medium (Biocorp, Poland) and antibiotics (chlortetracycline – 2 mg dm^-3^ and streptomycin – 30 mg dm^-3^) using dilution plate method. To isolate the fungi from wheat (Muszelka *var.)* the small pieces of plants were placed on a potato dextrose agar (PDA, Biocorp, Poland) in Petri dishes. The plates were then incubated for 10 days at 27°C. Fungal colonies were purified by repeated sub-culturing on PDA medium. The fungal isolates were identified as *Fusarium* sp. based on macroscopic and microscopic observations on PDA and Spezieller Nährstoffarmer Agar (SNA) media (Leslie and Summerell, 2006). The fungi were further cultured on PDA.

### Fungicides

The three different commercially available fungicides, qualified for three different groups of fungicides: triazoles, benzimidazoles and strobilurins were tested in the study. Each group of tested fungicides contained different substances as active compounds: tebuconazole, carbendazim, and azoxystrobin, respectively. The fungicides were based on three different mode of action affecting the fungi by: inhibiting specific enzymes in fungi that play a role in production of ergosterol necessary for the development of fungal cell walls, inhibiting mitosis and cell division and inhibiting respiratory processes of fungi, respectively.

### The Optimization of MT2 Microplates Approach

Traditionally MT2 microplates (Biolog^TM^) were used for the evaluation and identification of bacterial species based on their ability to utilize a range of different carbon sources (Kadali et al., 2012). In this study, the MT2 microplates were used as an alternative method for rapid assessment of the sensitivity of fungi to different fungicides. The appropriate composition of inoculating fluid for testing the sensitivity was selected. Three different types of inoculating fluids were tested: PM4-IF, PM9-IF and FF-IF. The inoculating fluids had the following composition: PM4-IF – Tween 40, Phytagel and D-glucose, PM9-IF – Tween 40, Phytagel, D-glucose and additive solution containing yeast nitrogen base and FF-IF – Tween 40 and Phytagel. The study included the optimization of the amount of mycelium and each fungicide added into the wells. The optimization step included the selection of appropriate concentrations of fungicides. The following four concentrations of each fungicides: 0.0005, 0.005, 0.05, 0.5% were tested. Aliquots of the individual fungicide suspension were loaded into the wells of the MT2 microplates. Two doses of fungicide 50 and 100 μl were tested while optimization. The wells were then inoculated with the resuspended fungal mycelium using 100 and 50 μl, respectively. To optimize method the *Fusarium* isolate G18/14 was selected. The optimization was prepared in triplicates. Appropriate positive and negative controls were provided for each isolate and each fungicide. The microplates were incubated at 27°C for 12 days and the optical density was monitored at 750 nm by every day readings using a microstation reader (Biolog^TM^).

### *Fusarium* Fungicides Resistance/Sensitivity Evaluation by MT2 Microplates Method

The assessment of *Fusarium* fungi resistance/sensitivity on selected fungicides was carried out using an alternative optimized method of MT2 microplates. The fungicides were tested at six concentrations: 0, 0.0005, 0.005, 0.05, 0.1, and 0.2%. The study included nine isolates of *Fusarium* described at *Fungal strains and growth conditions* section. The tested concentrations of each fungicide were applied in triplicate to the wells of MT2 microplates. Subsequently, the wells were inoculated with the mycelium of *Fusarium* suspended in PM4-IF inoculating fluid with glucose addition. To prepare the inoculum for MT2 microplates, the mycelial cells were harvested by loops from the surface of agar, homogenized and suspended in sterile inoculating fluid. Then, the suspension of each isolate was standardized into 75% of transmittance using turbidimeter (Biolog^TM^). Appropriate controls were set up for each isolate by loading the mycelium suspension into the wells without any fungicide and loading sterilized water instead. MT2 microplates were incubated at 27°C for 11 days. The optical density was monitored at 750 nm every day using a microstation reader (Biolog^TM^).

### *Fusarium* Fungicides Resistance/Sensitivity Evaluation by Hole-Plate Method

To verify the results of *Fusarium* fungicide resistance/sensitivity performed by MT2 microplates a hole-plate method based on zone of growth inhibition analysis was conducted. The fungicides were suspended in water as the following concentrations: 0, 0.0005, 0.005, 0.05, 0.5, 1, 2, 5, 10, 25, and 50% and afterward the fungicide sensitivity of the isolates were determined by assessing diameter of growth inhibition zones. *Fusarium* isolates (10-day-old cultures) were inoculated on 90 mm Petri dishes with PDA. Then, 8 mm diameter holes were cut in triplicates in PDA medium of each plate. After that, 100 μl of tested fungicides in each concentrations were added to individual holes. The study for each combination isolate-dose-fungicide were conducted in three replications. The results, as a diameter of growth inhibition zone, were collected after 1, 2, 3, and 4 days of incubation at 27°C.

### The Catabolic Profile of *Fusarium* Fungi Using FF Microplates

The utilization of particular substrates by each of the *Fusarium* isolates based on 95 low molecular weight carbon sources were assessed using the FF microplates (Biolog^TM^). The inoculation procedure was based on the original FF microplate (Biolog^TM^) method according to manufacturer’s protocol modified by Frąc (2012) and Janusz et al. (2015). To prepare inoculum, mycelia of each isolate were obtained by cultivation on PDA in the dark at 27°C through 10 days. After the suspension of the mycelium in inoculating fluid (FF-IF, Biolog^TM^) homogenization the transmittance was adjusted to 75% using a turbidimeter (Biolog^TM^). Hundred microliter of the above-mentioned mycelium suspension was added to each well and the inoculated microplates were incubated at 27°C through 10 days. The optical density at 750 nm was determined using a microplate reader (Biolog^TM^) every day, in triplicates. Functional diversity was determined by the number of different substrates utilized by the individual isolates and expressed as substrate richness (R). Phenotype profiles of *Fusarium* isolates were generated from FF microplates based on the growth intensity of the organism on particular substrates. Dendrogram was performed to show the correlation between the *Fusarium* isolates in relation to utilization of C-sources from the FF microplates.

### Statistical Analyses

Analysis of variance (ANOVA) was used to determine the differences in inhibiting effect of individual fungicides and their concentrations on the fungal isolates and in substrate richness of particular isolates. *Post hoc* analyses were performed using a Tukey test (HSD). The data were presented as 95% confidence intervals. Statistical significance was established at *p* < 0.05. Cluster analysis for substrate utilization, metabolic profiles and tested *Fusarium* isolates was used to detect groups in the data set and was made with Euclidian distance using Ward method approach. All statistical analysis was performed using Statistica software (version 10.0).

## Results

### The Optimization of MT2 Microplates Approach

**FIGURE 1:**
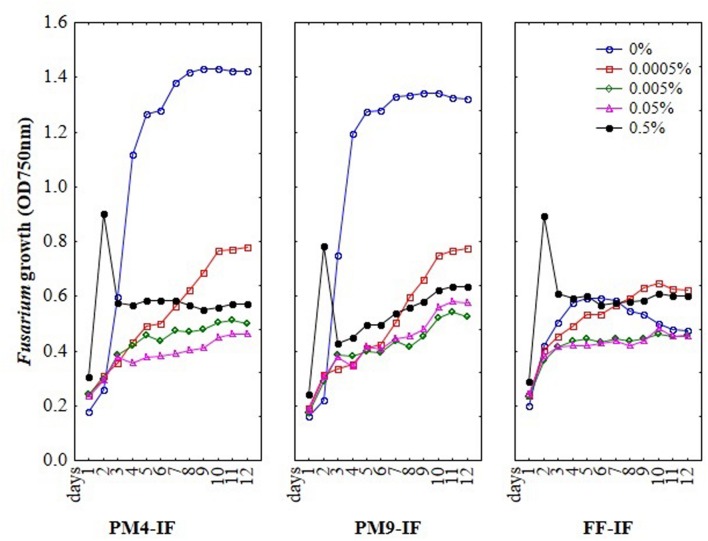
**The optimization results of inoculating fluids (IF) composition, fungicides concentrations and incubation time on the susceptibility of *Fusarium* using MT2 microplates method**.

To optimize MT2 method for evaluation *Fusarium* susceptibility to fungicides the various composition of inoculating fluids, different concentrations of fungicides and the ratio of mycelium to fungicide were tested. The assay was performed by addition of different concentrations of fungicides suspended in particular inoculating fluids into the wells of MT2 microplates. Further, the inoculum of *Fusarium* mycelia was added into each well and mixed by pipetting. Then, microplates were incubated for 11 days in the dark, at 27°C and optical density was measured every day. The trend in *Fusarium* growth during incubation time is presented at **Figure 1** and was mostly depended on used inoculating fluid.

**FIGURE 2:**
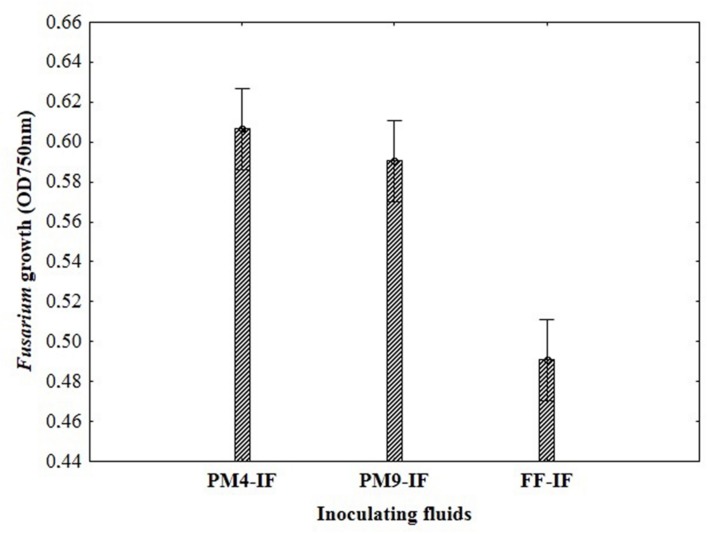
**The impact of different composition of inoculating fluids on the *Fusarium* growth in MT2 microplates with fungicides addition**.

**FIGURE 3:**
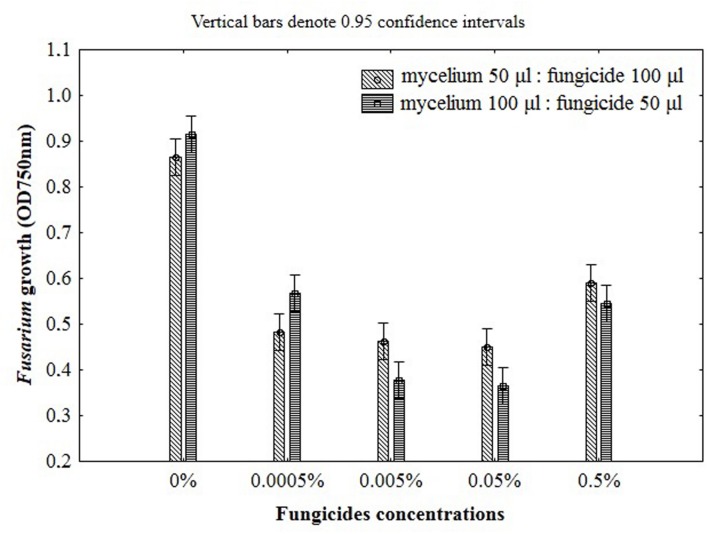
**The impact of fungicides concentrations and the ratio of mycelium to fungicide on the *Fusarium* susceptibility results using MT2 microplates method**.

To select a suitable inoculating fluid to evaluate fungal fungicides sensitivity/resistance, including the PM4-IF with glucose, PM9-IF with glucose and supplements and FF-IF known as standard inoculating fluid for filamentous fungi analysis were tested. Fungal growth was observed in all tested inoculating fluids, but in PM4-IF and PM9-IF was more intensive and significantly improved compared to FF-IF fluid (**Figure 2**). Moreover, in both inoculating fluids (PM4 and PM9) the fungal growth intensity in control wells (without fungicide addition) was significantly higher than in the wells with fungicides. Considering the fungal growth in FF-IF inoculating fluid, the growth intensity was very weak and almost at the same level for control wells with no fungicide addition and all tested fungicides concentrations (**Figure 1**). The highest differentiation between applied fungicides concentrations was found for the PM4-IF inoculating fluid, followed by the PM9-IF, whereas the lowest differentiation was obtained for the FF-inoculating fluid, suggesting significant influence of the inoculating fluid type on fungal growth, especially in stress conditions caused by fungicides presence. For this reason, the PM4-IF was selected after optimization for further study. The observed differences in fungal growth can be due to the inoculating fluid composition, but the transparency of fungicides (related to their color and consistency) might also have been responsible for observed artifacts, as the highest fungicides concentration (0.5%) was tested (**Figures 1** and **3**). If the dissolutions of fungicides causes optical density artifacts and to avoid such artifacts, those concentrations were omitted. Detailed the following lower concentrations were tested 0.1 and 0.2% (results described below).

The ratio between mycelium and fungicide volume in the microplates wells should also be defined in order to establish standard and controlled experimental conditions for assay. To select the best ratio of mycelial inoculum and fungicide the two schemes were used as follows: 50 μl of mycelium with 100 μl of fungicide and 100 μl of mycelium with 50 μl of fungicide. Growth inhibition became smaller with the increase of inoculum size, however, this effect was observed only for the lowest concentration of fungicide (0.0005%). Furthermore, the addition of 100 μl of mycelium suspension caused linear growth inhibition with increasing fungicides concentration (**Figure 3**). This effect was not observed for the ratio of 50 and 100 μl of mycelium and fungicide, respectively. Although the addition of 100 μl fungicides was twofold higher compared with the amount of 50 μl, the inhibition of fungal growth with increasing doses of fungicides was not clearly defined in this conditions, making difficult its precise measurement due to insufficient transparency of the mixture (**Figure 3**). For this reason, to properly determine the *Fusarium* growth, further approaches were decided to be performed with suspension containing a higher volume of mycelium (100 μl) and lower volume of fungicide (50 μl).

According to the results obtained, the *Fusarium* fungicide sensitivity/resistance assay was defined in PM4-IF inoculating fluid, using lower concentrations of fungicides (0.0005, 0.005, 0.05, 0.1, 0.2%) and the following 100:50 volume ratio of mycelium and fungicide. In order to assess and validate the usefulness and adequacy of the bioassay, the three fungicides (triazole, benzimidazole, and strobilurins) were tested against nine *Fusarium* isolates. The method was also validated in comparison to the traditional hole-plate zone of growth inhibition agar diffusion method.

### The Effect of Fungicides on Growth of *Fusarium* Strains Using MT2 Microplates Method

**FIGURE 4:**
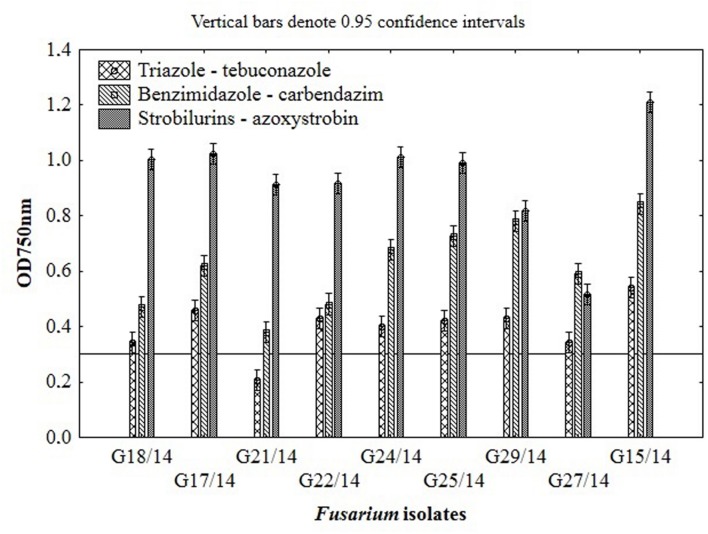
**The results of *Fusarium* isolates sensitivity/resistance on different fungicides using MT2 microplates method**.

**FIGURE 5:**
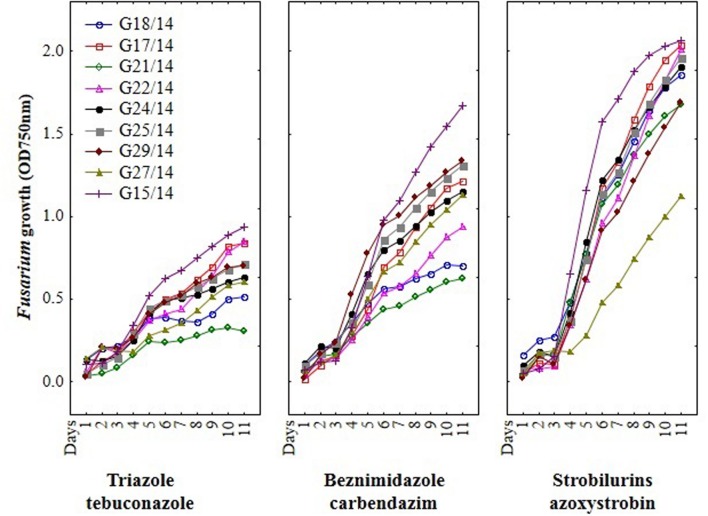
**The effect of three fungicides on the selected *Fusarium* isolates growth during eleven incubation days by MT2 microplates method**.

**FIGURE 6:**
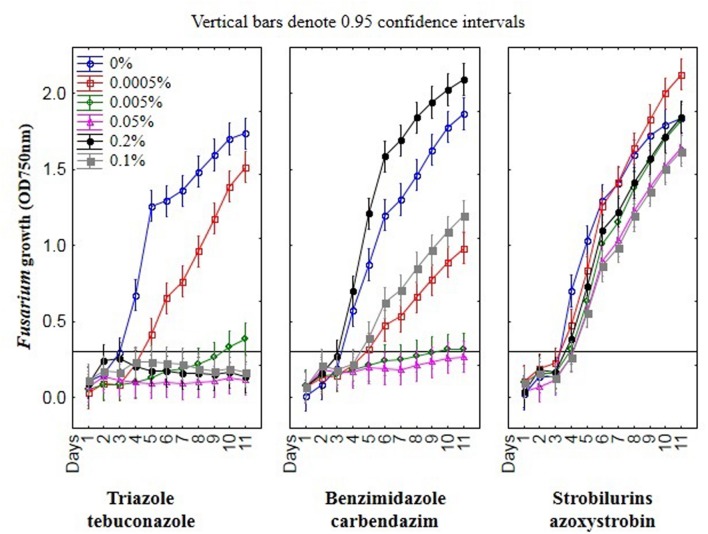
**The relationship between type of fungicide, doses of fungicide, action time of fungicide and *Fusarium* susceptibility expressed as growth intensity in MT2 microplates method**.

Among nine *Fusarium* isolates tested for sensitivity to three fungicides groups seven isolates showed high, one medium and one law resistance to strobilurins (azoxystrobin), five medium resistance to benzimidazole (carbendazim) and most of isolates were sensitive to triazole (tebuconazole) (**Figure 4**). The *Fusarium* susceptibility was dependent not only of isolates and used fungicide but also of the exposure time to toxic compound which is presented at **Figure 5**. The resistance to azoxystrobins could be connected with phenomena of AOX existing which was thoroughly discussed by Wood and Hollomon (2003). The most sensitive isolateto triazole- and benzimidazole-based fungicides was G21/14, whereas to strobilurins G27/14 (both soil isolates). The fungicide concentration, which inhibits mycelial growth by 50% relative to growth in unamended medium (EC50) was determined based on all readings of tested *Fusarium* isolates in particular days of analyses and presented as fungal growth intensity at **Figure 6**. The *Fusarium* growth inhibiting concentration of fungicides evaluated by MT2 microplates method was dependent on tested fungicide and their transparency, which might have been responsible for artifacts in measurements as false positive fungal growth. This effect was observed for benzimidazole-based fungicide applied at 0.1 and 0.2%. Although, all tested concentrations of tebuconazole caused fungal growth inhibition (determined as EC50), the *Fusarium* isolates were capable of good growth at 0.0005% tebuconazole concentration, and were severely inhibited by higher fungicide content (from 0.005 to 0.2%). The lowest tebuconazole concentration (0.0005%) inhibited fungal growth comparing to the control without fungicide, but with the increasing action time of tebuconazole, the fungi became resistant, which was expressed as increase of their growth. In the case of carbendazim four out of tested fungicide concentrations (0.0005, 0.005, 0.05, 0.1%) showed fungal growth inhibition. However, among above mentioned fungicide doses, the 0.1% concentration had the lowest value of EC50 which was connected with artifacts caused by low transparency of benzimidazole-based fungicide. The investigated *Fusarium* isolates demonstrated ability to tolerance azoxystrobin at all tested concentrations compare to the control without fungicide addition during 11 days of incubation (**Figure 6**).

Basing on the MT2 microplates method results, the highest fungal resistance was found for the azoxystrobin-based fungicide. The *Fusarium* isolates showed moderate resistance to carbendazim- and were sensitive to tebuconazole-based fungicides.

### The Effect of Fungicides on Growth of *Fusarium* Strains Using Hole-Plate Method

**FIGURE 7:**
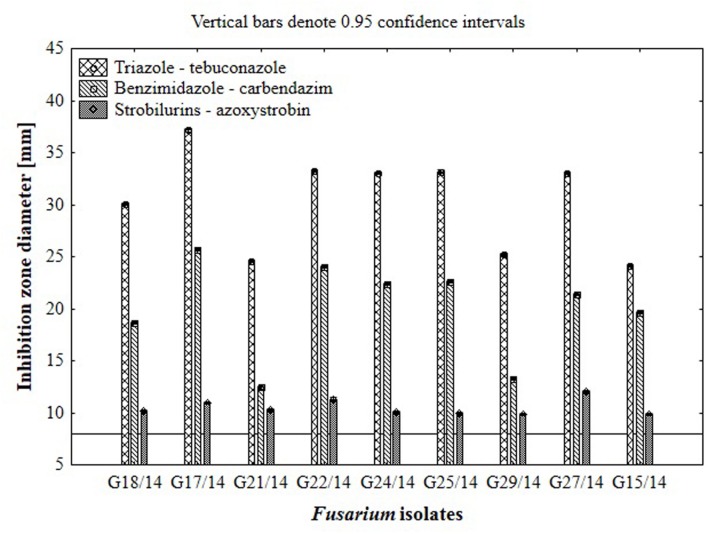
**The results of *Fusarium* isolates sensitivity/resistance on different fungicides using traditional hole-plate method**.

**FIGURE 8:**
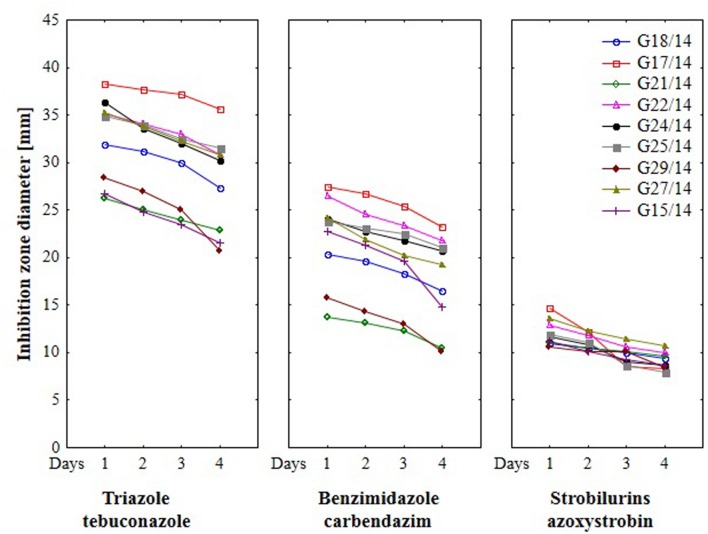
**The effect of three fungicides on the selected *Fusarium* isolates growth during four incubation days by traditional hole-plate method**.

**FIGURE 9:**
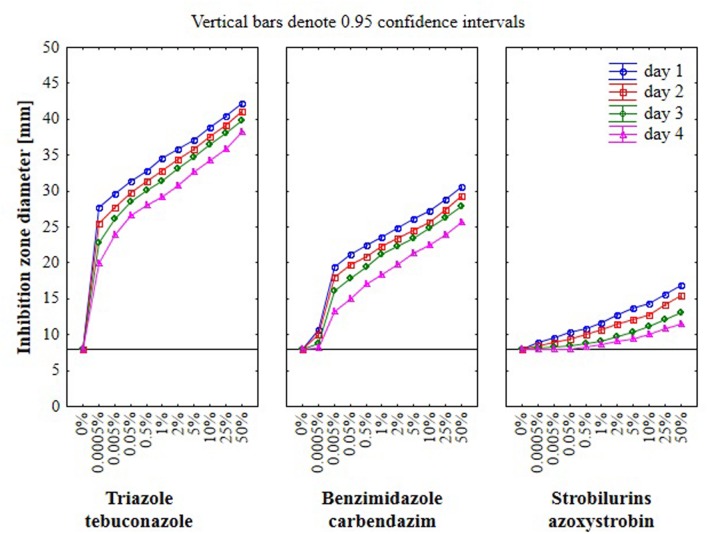
**The relationship between type of fungicide, doses of fungicide, action time of fungicide and *Fusarium* susceptibility expressed as inhibition zone diameter by traditional hole-plate method**.

**FIGURE 10:**
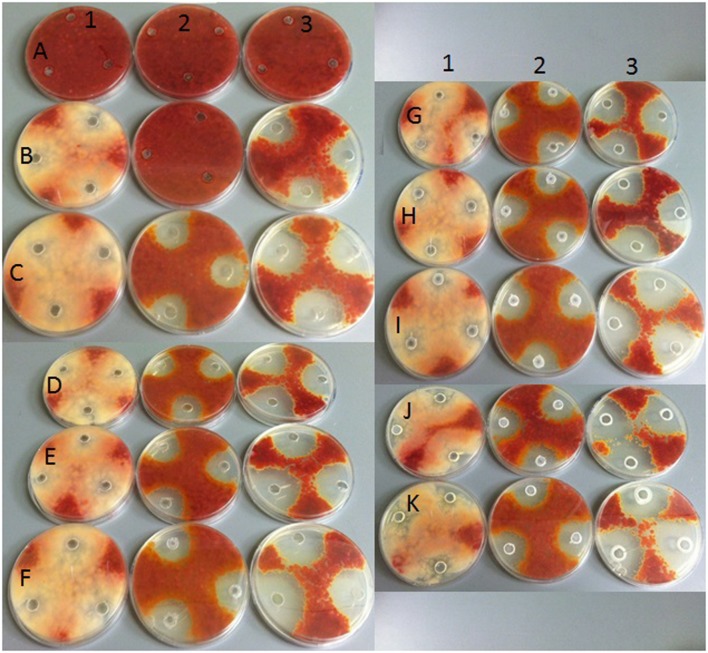
**Growth of selected Fusarium isolate on PDA with addition of various fungicides at different doses by hole plate method**. Explanations: in columns – fungicide type: (1) strobilurins – azoxystrobin; (2) benzimidazole – carbendazim; (3) triazole – tebuconazole; in rows fungicide concentrations: **(A)** 0%; **(B)** 0.0005%; **(C)** 0.005%; **(D)** 0.05%; **(E)** 0.5%; **(F)** 1%; **(G)** 2%; **(H)** 5%; **(I)** 10%; **(J)** 25%; **(K)** 50%.

Results from the conventional assay using hole-plate method showed that fungal growth of tested *Fusarium* isolates was strongly inhibited by azoxystrobin-based fungicide. However, the fungitoxicity of carbendazim and tebuconazole-based fungicides, expressed as diameter of fungal growth inhibition zone, was moderate and low, respectively (**Figure 7**). The inhibition zone diameter decreased during incubation time, indicating that all tested isolates were more or less able to grow in the presence of fungicide (**Figure 8**). The *Fusarium* resistance to azoxystrobin was observed at the same level for all tested isolates. The results indicated the differentiation of *Fusarium* sensitivity to tebuconazole and carbendazim depended on fungal isolate and time exposure to fungicide. The inhibition zone varied considerably among the isolates under the influence of triazole and benzimidazole-based fungicides during incubation time and no significant differences for growth inhibition was detected between isolates in the strobilurins presence. The *Fusarium* G17/14 isolated from wheat, showing the largest growth inhibition zones for both tebuconazole and carbendazim, differed significantly from all other isolates during all days of incubation. Both tebuconazole and carbendazim reduced fungal growth expressed as higher diameter of inhibition zone but tebuconazole provided the best fungal control (**Figures 9** and **10**). When tebuconazole was used the lowest (0.0005%) or above fungicide concentrations were defined as EC50 causing 50% increase of growth inhibition zone relative to control with water instead of fungicide addition. EC50 concentrations were 0.005% or above for carbendazim. Nevertheless, any of tested fungicides concentrations, even the highest (50%), cannot be defined as EC50 for azoxystrobin. With respect to sensitivity of *Fusarium* to fungicides tested in this work, we observed decrease of growth inhibition zone with increasing action time of fungicide for all concentrations, indicating that isolates adapted to fungicide exposition which can lead to development of fungal resistance to fungicides. The traditional hole-plate method can be used for testing both low and high fungicides concentrations.

The results indicated that similarly as in the MT2 microplates method the highest fungal resistance by hole-plate method was found for the azoxystrobin-based fungicide and moderate resistance for carbendazim. The *Fusarium* isolates were sensitive to tebuconazole-based fungicides.

### The Catabolic Profile of *Fusarium* Fungi Using FF Microplates

**FIGURE 11:**
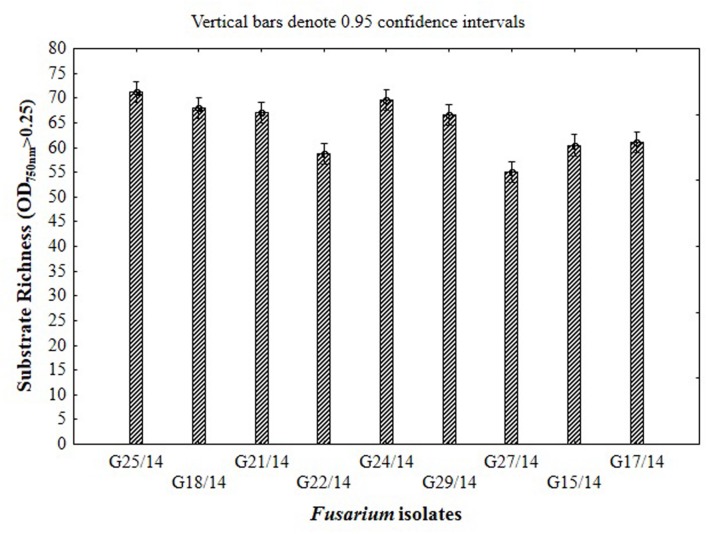
**Functional diversity of the *Fusarium* isolates expressed as substrate richness based on the capability of 95 C-sources utilization located in the wells of FF microplate**.

**FIGURE 12:**
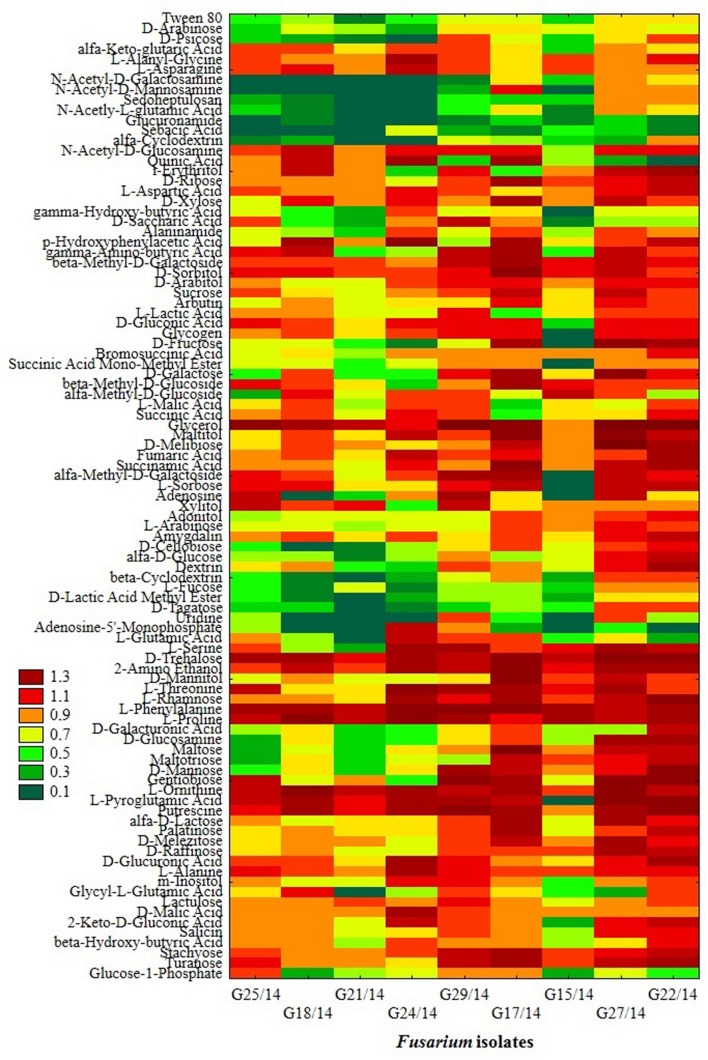
**Phenotype profiles of *Fusarium* isolates generated from FF microplates using Biolog System; color scale at the heat map indicates the growth of the organism (mycelial density A750 nm) in a particular substrate during 10 incubation days**.

**FIGURE 13:**
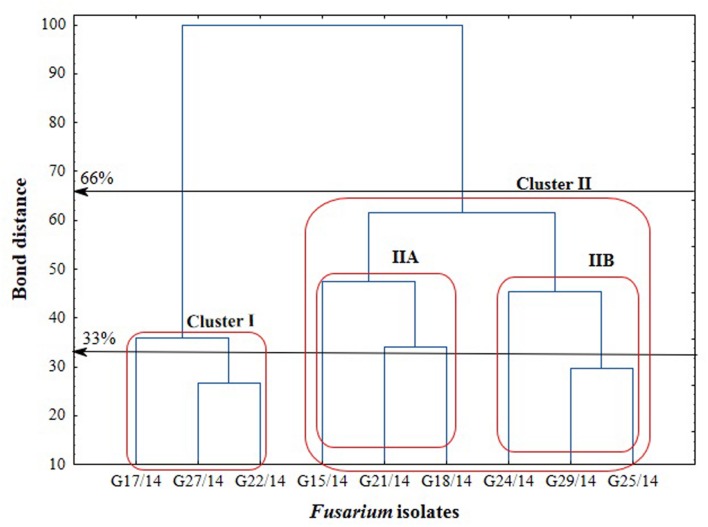
**Cluster analysis-based dendrogram showing correlation between the *Fusarium* isolates in relation to utilization of C-sources from the FF microplate**.

The application of the FF microplates allowed comparing the functional diversity of the nine *Fusarium* isolates. The substrate utilization abilities for the isolates tested revealed variability, indicating significant differences (up to 16 carbon sources) in the substrate richness values (**Figure 11**). However, all *Fusarium* isolates showed high catabolic activity, utilizing more than 50% of tested substrates. The highest capabilities to decompose of carbon sources were found for the following isolates G25/14, G24/14, G18/14, G21/14, and G29/14, which utilized 71, 70, 68, 69 and 69 out of 95 tested substrates, respectively. G17/14, G15/14, G22/14, G27/14 isolates were able to assimilate 61, 60, 59 and 55 out of 95 tested carbon sources, respectively (**Figure 11**). To determine the type of substrates that *Fusarium* isolates most utilized the optical density values indicating mycelial growth on the different carbon sources were subjected to cluster analysis and presented as heat map, according to the resulting growth (**Figure 12**). A detailed comparison of the carbon sources utilized by the *Fusarium* isolates revealed the most intensive differences in utilization of amino acids, carboxylic acids and carbohydrates. Among tested isolates three (G17/14, G22/14, G27/14) were able to utilized almost all among 95 tested carbon sources (**Figure 12**). Remaining isolates (G15/14, G18/14, G21/14, G24/14, G25/14, G29/14) in comparison to above-mentioned isolates were not capable of good growth at *N*-acetyl-D-galactosamine, *N*-acetyl-D-mannosamine, sedoheptulosan, *N*-acetyl-L-glutamic acid, beta-cyclodextrin, L-fucose, maltose and maltotriose. However, all isolates grew much better on amino acids and carbohydrates (L-alanine, L-ornityne, L-proline, L-phenylalanine, D-trehalose, turanose, D-sorbitol) than amides/amines and carboxylic acids (alaninamide, L-lactic acid, succinic acid). All isolates were unable to use glucuronamide and sebacic acid, although they utilized other substrates included into the groups of amines/amides and carboxylic acids (**Figure 12**). In cluster analysis two main distinct cluster groups were revealed. They included isolates which were capable of very intensive growth on most tested carbon sources (Cluster I) and isolates which utilized smaller number of tested substrates with less efficiency (Cluster II) (**Figure 13**). Cluster I contained two isolates from soil (G27/14, G22/14) grouped together and one isolate from plant (G17/14) as a separate branch. The clustered isolates were more sensitive to all tested fungicides than the rest of *Fusarium* strains. Cluster II assembled isolates, which also utilized large number of tested substrates but the intensity of their assimilation was significantly lower. This cluster was subdivided into two groups (IIA, IIB), of which subcluster IIA contained two the most sensitive isolates to tested fungicides (G18/14, G21/14) and one rather fungicides resistant isolate, as a separate branch. Subcluster IIB grouped two isolates which were able to cause the decomposition of dextrin and were not capable of good growth on D-xylose and one isolate decomposing D-xylose and not growing on dextrin, as a separate branch. Biolog FF analysis of the nine *Fusarium* isolates identified possible phenetic differences between the strains isolated from soils and plants with different fungicides sensitivity.

## Discussion

The monitoring of *Fusarium* with respect to fungicides resistance and sensitivity is important due to high quality food production. Traditional methods of qualitative and quantitative evaluation of *Fusarium* isolates resistance to fungicides are costly, time-consuming and could be harmful for environment due to usage of high amounts of potentially toxic chemicals (Pereira et al., 2013; Frąc et al., 2015). Therefore in this study approach to accurate and effective *Fusarium* fungicides resistance detection was designed and optimized using MT2 microplates (Biolog^TM^). MT2 microplates method has been described as screening and evaluation tool for identification and metabolic characterization of bacteria strains (Kadali et al., 2012; Taha et al., 2015). However, MT2 microplates method for fungal sensitivity have never been used before. This is the first study done using MT2 microplates (Biolog^TM^) to compare the efficacy of fungicides in controlling *Fusarium* isolates growth. The results of this study suggest that new MT2 microplates method can be used to determine *Fusarium* fungicides sensitivity/resistant efficiently, especially when low fungicides concentrations are tested. Using this method, *Fusarium* sensitivity to fungicides was best detected in PM4-IF inoculating fluid, for lower fungicides concentration and in the mixture 100 and 50 μl of inoculum and fungicide, respectively. The main features of optimized method are simplicity, cost efficiency and data reproducibility.

The efficacy of fungicides may change based on the level of resistance connected with the interactions between fungicides, cultivars and isolates (Amarasinghe et al., 2013). In our study, significant differences were found between the tested fungicides containing tebuconazole, carbendazim, or azoxystrobin, their concentrations and selected *Fusarium* isolates. According to our study the *Fusarium* isolates were highly sensitive to tebuconazole-based fungicide, which was indicated by both used methods (MT2 microplates, hole-plate agar). On the one hand, the obtained results are in agreement with other reports (Homdork et al., 2000; Matthies and Buchenauer, 2000; Amarasinghe et al., 2013), confirming the effectiveness of triazole fungicides in controlling *Fusarium* caused diseases. The results of Avozani et al. (2014) also indicated that *F. graminearum* isolates were susceptible to tebuconazole. On the other hand, triazole fungicides have been used as long as 30 years for controling fungal pathogens, therefore some of active substances have not been effective and emerging due to the lack of anti-resistance strategy (Spolti et al., 2014). A gradual reduction in *Fusarium* sensitivity to tebuconazole was observed in Germany. This effect concerned the isolates exposed to increasing fungicide doses (Klix et al., 2007). Tebuconazole-resistant *F. graminearum* isolates were also detected in China (Yin et al., 2009) and in United States (Spolti et al., 2014). Biological and agronomic factors such as the high metabolic and genetic diversity of the *Fusarium* pathogens and increasing use of triazole fungicides may pose a risk of selection of isolates that are both resistant to triazole fungicides and produce large amount of dangerous mycotoxins (Spolti et al., 2014). Contrary, in our study tebuconazole was the most effective among all tested fungicides. The disagreement between particular studies with our results may be due to differences in time exposure to fungicides and their concentrations. Moreover, when sublethal fungicides concentrations are used for long time the fungi can adapt to stress conditions and they become resistant to fungicides (Becher et al., 2010). Thus, the selection of fungicides concentrations plays a key role in the fungicides resistance development and mycotoxins production. In our study, a strong relationship between fungicides concentration and effectiveness of fungicides was observed. Further study on monitoring of *Fusarium* isolates resistant are crucial in order to better understand the risk for plant diseases and food security connected with fungi, which are less sensitive to triazole-based fungicides.

Although according to Avozani et al. (2014) azoxystrobin was the most potent fungicide to inhibit spore germination of five *F. graminearum* isolates, the results of our study showed high resistance to the fungicide containing this substance. This effect was confirmed by both new MT2 microplates and traditional hole-plate methods. Limitation of strobilurin effectiveness can be caused by AOX once an infection is established, but AOX is unable to interfere significantly with strobilurin action during germination (Wood and Hollomon, 2003). Audenaert et al. (2010) reported that sublethal doses of fungicides can induce hydrogen peroxide as catalyser of toxin deoxynivalenol biosynthesis and can cause the development of fungal resistance and adaptation to the fungicide influence (Becher et al., 2010). For these reasons some fungicides treatment can cause increase of mycotoxins, which was found after strobilurin-based fungicides application (Pirgozliev et al., 2002). However, to date there has not been sufficient evidence to explain the role of fungicides in increasing mycotoxins in infected plants (Amarasinghe et al., 2013). Our study indicated that most of *Fusarium* isolates were resistant to azoxystrobin, which represent the group of strobilurin-based fungicides.

In this work we addressed the potential of new MT2 microplates method in *Fusarium* fungicides sensitivity assessment and studied the catabolic profiles of tested isolates. Further studies are needed to evaluate an adaptation of *Fusarium* isolates to fungicides exposure by changes of carbon sources utilization and phenomic profiles. Becher et al. (2010) indicated that exposure to tebuconazole resulted in the emergence of two morphologically separate phenotypes differing in the degree of tebuconazole resistance, fitness, virulence and mycotoxins production. Due to the prelevance of fungicides in the control of *Fusarium* pathogens it is important to assess and monitor the isolates fungicides sensitivity and investigate the mechanisms leading to fungicides resistance.

## Conclusion

This study demonstrated that the MT2 microplates (Biolog^TM^) method developed here represents a cheap (less materials are used), environmentally friendly (significant reduction of amount of used fungicides, compering to hole-plate method), not as much time-consuming as for hole-plate approach) and effective tool for the rapid fungicides sensitivity/resistance assessment of *Fusarium* isolates. Results from traditional hole-plate method based on zone of growth inhibition and MT2 microplates method were summarized. The substantial relationship between results obtained using both methods was found, what clearly indicate that MT2 microplates method might be used successfully vice traditional hole-plate technique. Comparing the results of the traditional hole-plate and MT2 microplates methods we found strong relationship in whole *Fusarium* fungicide resistance, although there were some differences with respect to individual fungal isolates.

All tested fungicides showed toxicity for mycelium growth, but this effect was dependent on type, concentration and action time of fungicide and *Fusarium* isolate. The tebuconazole was most potent, providing increased efficiency in the growth inhibition of all tested isolates. Nevertheless, the isolates showed different sensitivity to the fungicide concentration. The carbendazim caused *Fusarium* growth inhibition, but some of isolates were resistant to this fungicide. Almost all among tested isolates were resistant to azoxystrobin-based fungicide.

The sensitivity determined in this study constitutes a critical step for monitoring changes in metabolic profile and identification of risk factors related to increase or decrease in particular carbon sources utilization intensity leading to decline in sensitivity.

## Author Contributions

Conceived and designed the experiments: MF, AG, KO, NK. Performed the experiments: MF, AG, KO, NK. Analyzed the data: MF, AG, KO, NK. Contributed reagents/materials/analysis tools: MF, AG, KO, NK. Wrote the paper: MF, AG, KO, NK.

## Conflict of Interest Statement

The authors declare that the research was conducted in the absence of any commercial or financial relationships that could be construed as a potential conflict of interest.
